# Extracellular Vesicles Released from Neprilysin Gene-Modified Human Umbilical Cord-Derived Mesenchymal Stem Cell Enhance Therapeutic Effects in an Alzheimer's Disease Animal Model

**DOI:** 10.1155/2021/5548630

**Published:** 2021-12-03

**Authors:** HyeJu Jeong, Ok Joon Kim, Seung-Hun Oh, Sanghoon Lee, Han A. Reum Lee, Kee Ook Lee, Boo-Yong Lee, Nam Keun Kim

**Affiliations:** ^1^Department of Neurology, CHA Bundang Medical Center, CHA University, Seongnam, Gyeonggi 13496, Republic of Korea; ^2^Department of Food Science and Biotechnology, College of Life Science, CHA University, Seongnam, Gyeonggi 13488, Republic of Korea; ^3^Department of Biomedical Science, College of Life Science, CHA University, Seongnam, Gyeonggi 13496, Republic of Korea

## Abstract

Alzheimer's disease (AD) animal studies have reported that mesenchymal stem cells (MSCs) have therapeutic effects; however, clinical trial results are controversial. Neprilysin (NEP) is the main cleavage enzyme of *β*-amyloid (A*β*), which plays a major role in the pathology and etiology of AD. We evaluated whether transplantation of MSCs with NEP gene modification enhances the therapeutic effects in an AD animal model and then investigated these pathomechanisms. We manufactured NEP gene-enhanced human umbilical cord-derived mesenchymal stem cells (hUC-MSCs) and intravenously transplanted them in A*β*_1-42_-injected AD animal models. We compared the differences in behavioral tests and immunohistochemical assays between four groups: normal, A*β*_1-42_ injection, naïve hUC-MSCs, and NEP-enhanced hUC-MSCs. Both naïve and NEP-enhanced hUC-MSC groups showed significant improvements in memory compared to the A*β*_1-42_ injection group. There was no significant difference between naïve and NEP-enhanced hUC-MSC groups. There was a significant decrease in Congo red, BACE-1, GFAP, and Iba-1 and a significant increase in BDNF, NeuN, and NEP in both hUC-MSC groups compared to the A*β*_1-42_ injection group. Among them, BDNF, NeuN, GFAP, Iba-1, and NEP showed more significant changes in the NEP-enhanced hUC-MSC group than in the naïve group. After stem cell injection, stem cells were not found. Extracellular vesicles (EVs) were equally observed in the hippocampus in the naïve and NEP-enhanced hUC-MSC groups. However, the EVs of NEP-enhanced hUC-MSCs contained higher amounts of NEP as compared to the EVs of naïve hUC-MSCs. Thus, hUC-MSCs affect AD animal models through stem cell-released EVs. Although there was no significant difference in cognitive function between the hUC-MSC groups, NEP-enhanced hUC-MSCs had superior neurogenesis and anti-inflammation properties compared to naïve hUC-MSCs due to increased NEP in the hippocampus by enriched NEP-possessing EVs. NEP gene-modified MSCs that release an increased amount of NEP within EVs may be a promising therapeutic option in AD treatment.

## 1. Introduction

Alzheimer's disease (AD) is the most common form of dementia that induces the deterioration of active daily life. AD is pathologically characterized by extracellular deposits (senile plaques) of *β*-amyloid (A*β*) and intracellular neurofibrillary tangles, leading to selective neuronal loss in the diseased brain [1–3]. Progressive memory loss and cognitive decline occur as the disease progresses [2–4]. The main treatments for AD approved by the FDA include acetylcholinesterase inhibitors and N-methyl D-aspartate receptor antagonists; these drugs do not affect AD pathophysiology, but rather offer temporary symptomatic relief [5–7]. Numerous studies have been conducted each year focusing on the development of a disease-modifying treatment of AD, but the results are somewhat unsatisfactory. Since AD is complex in nature and affects multiple pathways and regions, a single small-molecule approach may not provide substantial benefit in the absence of other interventions [8, 9]. Therefore, cell therapy with multiple mechanisms of treatment has emerged as a therapeutic strategy for the treatment of AD [7, 10–15].

Many studies have been conducted for therapeutic purposes using various stem cell origins in AD animal models. Embryonic stem cells, neural stem cells (NSCs), induced pluripotent stem cells, and mesenchymal stem cells (MSCs) are used in this regard [7, 14, 16–19]. Among these, MSCs have been the most commonly investigated. Although experimental studies in AD have demonstrated some measures of success, MSC therapies in clinical trials have not resulted in widespread success [15, 20, 21]. To enhance their properties and overcome these limitations, MSCs have been modified by exogenous gene transfer [22]. MSCs are relatively easy to isolate, differentiate into a wide variety of cell types, exhibit extensive expansion in culture without a loss of differentiative capacity, and possess hypoimmunogenicity and immunosuppression, anti-inflammatory properties, and homing to damaged tissues [12, 23, 24]. Therefore, MSCs are a superior cell source for therapeutic transgene expression to produce various cytokines for engineered MSC therapy [25, 26].

Among the various causes of AD, A*β* peptides play an important role in the pathogenesis of AD [2]. Secreted enzymes, which have an affinity for specific domains within the A*β* amino acid sequence and the ability to cleave these peptides to shorter, more benign forms, are critical for the catabolism of circulating A*β* [27]. These proteins include the insulin-degrading enzyme, neprilysin (NEP), and its homologue endothelin-converting enzyme, angiotensin-converting enzyme, matrix metalloproteinase-9, and plasmin [27, 28]. Among these, NEP has been shown to play a major role in the clearance of A*β* in the brain [28–30]. NEP, a zinc metallopeptidase, is widely expressed in many tissues, especially in the brush border of intestinal and renal tubular epithelial cells [31–33]. In the central nervous system, NEP is mainly expressed by neurons, activated astrocytes, and microglia [34–36]. In neurons, NEP is subcellularly localized along axons and synapses [36, 37], where NEP-mediated A*β* degradation mainly occurs [36, 38–40]. Therefore, NEP may be an attractive therapeutic target for AD. In this study, we investigated whether NEP-enhanced human umbilical cord-derived mesenchymal stem cells (hUC-MSCs) could lead to a further decrease in pathogenic accumulation of A*β* and then more alleviation of cognitive dysfunction than naïve hUC-MSCs in an A*β*_1-42_-injected AD mouse model. We also evaluated whether these effects of hUC-MSCs were induced by the penetration of stem cells or stem cell-released extracellular vesicles (EVs).

## 2. Materials and Methods

### 2.1. Animal

Male ICR (Institute of Cancer Research) mice (30~35 g, 7 weeks old) purchased from Orient Bio (Seongnam, Gyeonggi, Korea) were used. Mice were housed in rooms with a humidity of 60 ± 10% and a temperature of 23°C with repeated 12 h light-dark cycles. Food and water were allowed freely. Behavioral experiments were conducted during daylight. The animals had an adaptation period of 1 week to adjust to the laboratory before the experiment. The use and management of experimental animals followed the guidelines provided by the Institutional Animal Care and Use Committee of CHA University (IACUC nos. 190094 and 200061).

### 2.2. Preparation of Intrahippocampal A*β*_1-42_-Injected AD Model

Synthetic peptide A*β*_1-42_ (Merck Millipore, Burlington, MA, USA) was dissolved in a 1% acetic acid solution to a concentration of 400 *μ*M. After aliquot, A*β*_1-42_ was stored at -20°C before use. The A*β*_1-42_ solution was incubated at 37°C for 3 days before use. The operational procedure was described in detail in a previous our study [41]. Briefly, mice were anesthetized with anesthetic agents (40 mg/kg tylamine/zolazepam (Zoletil™) and 5 mg/kg xylazine) and fixed with a stereotaxic frame (Kopf Instruments, Tujunga, CA, USA) for surgery. A*β*_1-42_ peptide (400 *μ*M) was injected into bilaterally in the hippocampus (-2.0 mm front/back, ±1.3 mm middle/side, and -2.2 mm back/side to bregma). The A*β*_1-42_ solution was slowly injected into the injection site at a rate of 1 *μ*L/min; each mouse received 2 *μ*L of the solution.

### 2.3. hUC-MSC Culture

hUC-MSCs were purchased from CHA Biotech (Seongnam, Korea); the characteristics of the cells have been described in detail in the previous studies [42–45]. hUC-MSCSs were cultured in low-glucose minimum essential medium (Gibco, Gaithersburg, MD, USA) supplemented with 10% fetal bovine serum (Gibco, Gaithersburg, MD, USA), 50 *μ*g/mL gentamycin (Sigma-Aldrich, St. Louis, MO, USA), 1 *μ*g/mL heparin (Sigma-Aldrich, St. Louis, MO, USA), and 25 ng/mL fibroblast growth factor-4 (Peprotech, Rocky Hill, NJ, USA). Seven or nine passage cells were used for the experiments.

### 2.4. Construction of NEP Gene-Modified hUC-MSCs

pEGFP-neprilysin-Neo/kanamycin-resistant gene cDNA clone plasmid was purchased from Cosmogenetech (Seoul, Korea). The plasmid DNA containing the kanamycin resistance gene, NEP, and green fluorescent protein (GFP) gene was transformed into HIT competent cell T-DH5a (RBC Bioscience, New Taipei, Taiwan). Competent cells were proliferated in Bacto agar medium (BD Difco™, Bergen County, NJ, USA) and LB broth (BD Difco™, Bergen County, NJ, USA) containing kanamycin. The transformation plasmid DNA was extracted using the NucleoBond Xtra Midi Plus Kit (Macherey-Nagel, Bethlehem Township, PA, USA). The extracted plasmid DNA was reacted with Lipofectamine Stem Reagent (STEM00015, Invitrogen, USA) and Opti-MEM™ I Reduced Serum Medium (Gibco, Gaithersburg, MD, USA) and then added to the culture medium in which hUC-MSCs grew. To confirm the transfection of cells after 2 days, the level of GFP was observed using a Carl Zeiss Axiovert 200M microscope (Carl Zeiss, Baden-Württemberg, Germany).

### 2.5. Quantitative Real-Time Polymerase Chain Reaction (qRT-PCR) for NEP Gene Expression

RNA extracted from cells using TRIzol Reagent (Ambion, Austin, TX, USA), chloroform, and isopropanol was quantified using an FC-1100 NanoReady spectrophotometer (Lifereal, Hangzhou, China). The same amount of RNA obtained from each sample was added to SuPrimeScript RT Premix (GeNet Bio, Chungnam, Korea), mixed, and then incubated at 50°C for 60 min and at 70°C for 10 min. The reaction was carried out for 40 cycles using the Biosystems ViiA 7 Real-Time PCR System with TOPreal qPCR 2X PreMIX SYBR Green (Enzynomics, Daejeon, Korea) and primers. The following primers were used: GAPDH: 5′-AGCAATGCCTCCTGCACCACCAAC-3′ (forward) and 5′-CCGGAGGGGCCATCCACAGTC-3′ (reverse) and NEP: 5′-TCTTGTAAGCAGCCTCAGCC-3′ (forward) and 5′-ACATAAAGCCTCCCCACAGC-3′ (reverse). NEP levels were normalized against those of GAPDH, and amplifications were independently replicated four times on different days.

### 2.6. Western Blot

Naïve and NEP-enhanced hUC-MSCs were lysed with PRO-PREP™ Protein Extraction Solution (INtRON, Seongnam, Korea). Lysed cells were centrifuged at 13000 rpm at 4°C for 15 min to obtain a protein in the supernatant. The protein quantification was performed by using the Pierce™ BCA Protein Assay Kit (Thermo Fisher, Waltham, MA, USA), incubated at 37°C for 30 min, and absorbance was measured at 562 nm with a set of albumin standards. Equal amounts of protein (50 *μ*g) were loaded onto a 10% SDS gel, electrophoresed, and transferred to Immobilon-P PVDF membranes (Merck Millipore, Burlington, MA, USA). The protein was reacted with *β*-actin antibody (1 : 1000; Santa Cruz, TX, USA) or CD10 (NEP) antibody (1 : 1000; Santa Cruz, TX, USA) and then incubated with goat anti-mouse immunoglobulin (Ig) G antibody (HRP) (1 : 5000; Genetex, Irvine, CA, USA). The membrane was then washed and reacted using a Clarity Western ECL substrate (BIO-RAD, Hercules, CA, USA). Proteins were detected using an ImageQuant LAS 4000 instrument (GE Healthcare, Chicago, IL, USA). Experiments were independently replicated four times on different days. The detected protein was quantified by measuring the density using ImageJ.

### 2.7. Transplantation of hUC-MSCs

**Figure 1:**
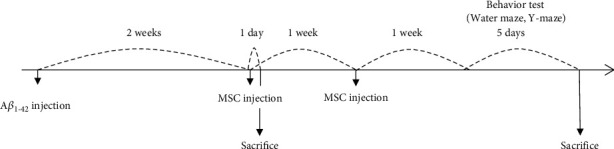
Experimental schedule. Two weeks after A*β* injection into the bilateral hippocampus, stem cells were injected twice at intervals of 1 week. A week after last injection of stem cells, various behavior tests and immunohistochemical analyses were performed. EV study was performed on a day after first stem cell injection. A*β*: *β*-amyloid; MSC: mesenchymal stem cell; EV: extracellular vesicle.

The in vivo experimental design is shown in Figure 1. First, animals were randomly divided into four groups (*n* = 10/group) for behavioral and immunofluorescence analyses: a normal group, a group that was injected with A*β*_1-42_ (A*β* group), a group that was injected with naïve hUC-MSCs after A*β*_1-42_ injection (MSC group), and a group that was injected with NEP-enhanced hUC-MSCs after A*β*_1-42_ injection (NEP-MSC group). The injection procedure was the same for all the groups. hUC-MSCs (5 × 10^5^) were injected into the tail vein using 31-gauge insulin syringes (injection rate: 0.03 mL/min) on the second and third weeks after A*β*_1-42_ injection. Before transplantation of cells, 0.25 mL of 20% mannitol (Sigma, St. Louis, MO, USA) in phosphate-buffered saline was injected to disrupt the preinjection blood-brain barrier (BBB) [46]. No immunosuppressive drugs were administered.

### 2.8. Behavioral Tests

We performed the Morris water maze and Y-maze tests to evaluate memory following the schedule presented in Figure 1.

#### 2.8.1. Morris Water Maze Test

The water maze used a circular pool filled with water (diameter: 90 cm and height: 55 cm; 242727 CC). The escape platform (diameter 6 cm and height 29 cm) was placed at the center of one of the four quadrants in the maze, 1 cm below the surface of the water. The training phase was conducted three times per day for 4 consecutive days. On the first day of the study, the mice were allowed to swim freely in the maze for 60 s to adjust to the maze. On the first day of the training stage, the platform was placed so that it was visible above the surface of the water, and for the next 3 days, the platform was placed so that it was not visible below the surface of the water; the position of the platform remained constant, and in each training experiment, mice swam for 60 s or until they found the platform. Each mouse was then placed on the platform for 5 s when it found the platform and for 20 s when it did not. A SMART 3.0 video tracking system (Panlab Harvard Apparatus, Barcelona, Spain) was used to record the total escape time and the time remaining in the target area. To evaluate the memory of mice, a probe test was conducted on day 5. During these probe tests, the platform was removed from the maze, allowing the mice to swim freely for 60 s in the maze. The time spent in the quadrant where the platform was located was recorded, indicating how well the mouse remembered the location of the platform.

#### 2.8.2. Y-Maze Test

The Y-maze test was conducted to evaluate the operational memory changes in mice. The three arms that make up the Y-maze were 30 cm long, 10 cm wide, and 20 cm high. Each arm was placed at an angle of 120° to the other arm. The mice were placed in the center of the Y-maze and allowed to move freely for 5 min. The input sequence of all the experiments was recorded. Spontaneous alternations were calculated by dividing the three consecutive arm input sequences by (total arm input-2). A low alternation meant that the mouse did not remember the arm it just visited and suggested a memory disorder.

### 2.9. Brain Histology

After the water maze test, mouse brain samples were prepared for Congo red staining and immunofluorescence. Mice were euthanized with carbon dioxide and transcardially perfused with 4% formaldehyde (Junsei Chemical, Tokyo, Japan). Perfused brains were separated from the skull and fixed with 4% formaldehyde overnight at 4°C. After fixation, brains were soaked in 30% sucrose solution for dehydration and incubated at 4°C until the brain tissue sank. The brains protected from cryogenic conditions were frozen in an optimal cutting temperature compound and stored at -80°C. Brain tissue was sectioned at a thickness of 20 *μ*m in a cryostat using a cryostat microtome (Leica CM3050, Wetzlar, Germany). Cells stained with Congo red dye, and immunohistochemistry techniques were observed around the hippocampus in 4–5 mice per group using an eXcope T300 optical microscope (Olympus, Tokyo, Japan) or a fluorescence microscope.

### 2.10. Congo Red Staining

Congo red staining was performed to confirm the presence of A*β* plaques in the hippocampus. The sections were stained with hematoxylin Quick stain (Sigma-Aldrich, St. Louis, MO, USA). Briefly, sections were washed in distilled water for 30 s and incubated for 20 min in an alkaline saturated sodium chloride solution. After removing the solution, the sections were incubated for 1 h in Congo red solution. Subsequently, they were rinsed with water and mounted. The amyloid dyed in Congo red was observed under an eXcope T300 optical microscope.

### 2.11. Immunofluorescence

Brain tissue sections were rinsed for 10 min with 0.2% Triton-X 100 and incubated for 30 min with 5% normal goat serum (Vector Laboratories, Burlingame, CA, USA). Sections were then incubated with primary antibodies against beta secretase-1 (BACE-1) (1 : 100, Abcam, Cambridge, UK), brain-derived neurotrophic factor (BDNF; 1 : 500; Abcam, Cambridge, UK), nerve nuclei (NeuN) (1 : 100; Millipore, MA, USA), glial fibrous acid protein (GFAP) (1 : 100, BD Bioscience Bergen County, NJ, USA), ionized calcium-binding adapter molecule-1 (Iba-1; 1 : 500; Wako Pure Chemical Co., Kyoto, Japan), NEP (CD10, 1 : 100, Santa Cruz, TX, USA), STEM121 Human Cytoplasmic Marker (1 : 500, Cellartis, Kyoto, Japan), CD63 (1 : 200, Abcam, Cambridge, UK), and CD81 (1 : 200, Abcam, Cambridge, UK). The sections were then incubated with goat anti-mouse Ig G antibody combined with Alexa 488 and 555 (1 : 200; Invitrogen, CA, USA) or goat anti-rabbit Ig G antibody combined with Alexa 488 (1 : 200; Invitrogen, Waltham, CA, USA) for 2 h and stained with 4′,6-diamidine-29-phenylindole dihydrochloride (DAPI; Vector Laboratories, Burlingame, CA, USA). The amounts of BACE-1, GFAP, and NEP were analyzed using an Axiovert 200M microscope (Carl Zeiss, Oberkochen, Germany). The expression of NeuN, BDNF, Iba-1, STEM121, CD63, and CD81 proteins was analyzed using an ECLIPSE Ni-U microscope (Nikon, Tokyo, Japan). To identify injected cells and secreted EVs within brain parenchyma, the samples of STEM121, CD63, and CD81 were used the day after the 1st injection of stem cells. All immunohistochemical analyses were conducted using four or more samples from each group.

### 2.12. DNA Extraction and qRT-PCR for Human-Specific Gene Alu Sequences

To detect the human cells within the mouse brain after intravenous injection of hUC-MSCs, we performed qRT-PCR for human-specific gene Alu sequences. The samples were taken from three mouse brain tissue samples per group, using 25 mg of hippocampus tissue. hUC-MSCs were used as a positive control. DNA was extracted using the QlAamp DNA Mini Kit (Qiagen, Hilden, Germany) and quantified as FC-1100 NanoReady (Lifereal, Hangzhou, China). The qRT-PCR was progressed using 25 ng of DNA. The reaction was carried out for 40 cycles using the Biosystems ViiA 7 Real-Time PCR System with TOPreal qPCR 2X PreMIX SYBR Green (Enzynomics, Daejeon, Korea). The following primers were used: human-specific Alu: 5′-CATGGTGAAACCCCGTCTCTA-3′ (forward) and 5′-GCCTCAGCCTCCCGAGTAG-3′ (reverse). Human-specific Alu sequence expression in the mouse brain tissue of each group was expressed as a cycle threshold (Ct) value. Also, to calculate the number of human cells in mouse brain tissue based on the Ct value, we generated the equation by using independent samples as reported in a previously published study [47]. We produced the mixed populations by diluting hUC-MSCs to the cells of the normal mouse (hUC-MSCs compared to normal mouse cells: 100%, 50%, 5%, 0.5%, and 0%, and the total number of cells remains constant at 5 × 10^5^). We performed qRT-PCR for human-specific Alu sequences with 25 ng of DNA extracted in dilution groups and generated an Alu standard curve (calculation curve) and equation [48]. Subsequently, we estimated the number of human cells contained within mouse brain in each group by substituting the Ct value of the experimental group sample in the standard curve and equation. The detection limit was 0.005% of hUC-MSCs in the total mixture population [47].

### 2.13. EV Isolation

Conditioned media were collected on the second day after the transfection. The media was centrifuged for 30 min at 2000 g to remove dead cells. After taking the supernatant, we added 0.5 volumes of the Total Exosome Isolation Reagent (Invitrogen, Waltham, CA, USA) and incubated overnight at 4°C. Afterwards, centrifugation was performed at 10000 g for 1 h at 4°C, the supernatant was discarded, and western blotting and qRT-PCR experiments were conducted using EV pellets. Protein (70 *μ*g) was used in the western blot for protein analysis of EVs. These experiments were repeated five times.

## 3. Statistical Analysis

The experiment was statistically analyzed using the SPSS 18.0 (SPSS Inc., Chicago, IL, USA) program for Windows. Results were compared with repeat measurements, one-way analyses of variance (ANOVA), and post hoc analysis using the Holm–Bonferroni method. A *t*-test was used to compare the two groups. All data were expressed as the mean ± standard error of the mean (SEM). Statistical significance was set at *P* < 0.05.

## 4. Results

### 4.1. Transfection of NEP Plasmid DNA to hUC-MSCs Increased NEP Gene Expression and Protein

**Figure 2:**
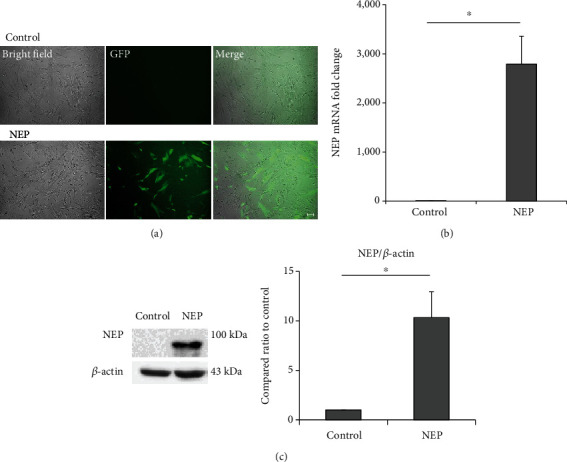
Increased NEP gene expression and protein level in hUC-MSCs after NEP gene transfection. (a) GFP fluorescence image of MSCs on the second day after plasmid DNA transfection. Scale bar = 100 *μ*m. (b) The relative amount of NEP mRNA expression in naïve and NEP-enhanced hUC-MSCs in the qRT-PCR experiment after gene transfection. (c) The amount of NEP protein assessed by western blot after the transfection. The bar on the graph represents the mean ± SEM. Data were analyzed by use of *t*-test. ^∗^*P* < 0.05. hUC-MSC: human umbilical cord-derived mesenchymal stem cell; GPF: green fluorescent protein; NEP: neprilysin; qRT-PCR: quantitative real-time polymerase chain reaction; SEM: standard error of the mean.

Plasmid DNA containing NEP and GFP gene sequences was inserted into the hUC-MSCs to construct NEP-enhanced hUC-MSCs. Two days after transfecting plasmid DNA in cells, GFP manifestations of cells were observed with fluorescent microscopes to confirm the insertion of genes. As a result, unlike the naïve hUC-MSC group, cells exhibiting green fluorescence were observed in the hUC-MSC group treated with NEP-GFP plasmid DNA (Figure 2(a)). In addition, qRT-PCR and western blot analyses were conducted to determine whether such transfection actually increased the expression of NEP genes and proteins in the NEP-enhanced hUC-MSC group. The hUC-MSC group treated with NEP-GFP plasmid DNA had much higher NEP gene expression than naïve hUC-MSCs (*P* = 0.0162) (Figure 2(b)), and the amount of protein increased approximately tenfold (*P* = 0.0381) (Figure 2(c)).

### 4.2. Transplantation of Naïve and NEP-Enhanced hUC-MSCs Improved Memory in A*β*_1-42_-Injected AD Mice

**Figure 3:**
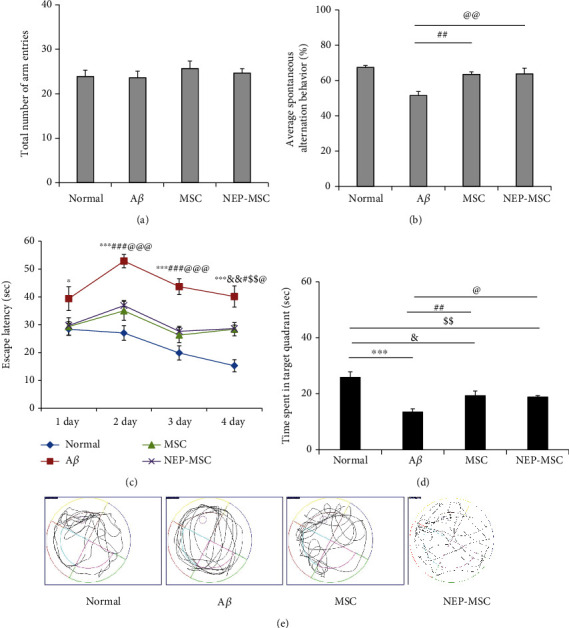
Behavioral tests through Y-maze and Morris water maze. (a) The number of total arm entries and (b) spontaneous alternations in the Y-maze test. Escape latency during the (c) four-day training period and on the (d) last day and the swimming path of the mouse recorded during the (e) probe test in Morris water maze test. Data represent the mean ± SEM and were analyzed by one-way ANOVA. ^∗∗∗^*P* < 0.001 comparison between A*β* and normal; ^#^*P* < 0.05 and ^###^*P* < 0.001 comparison between MSC and A*β*; ^@^*P* < 0.05 and ^@@@^*P* < 0.001 comparison between NEP-MSC and A*β*; ^$$^*P* < 0.01 comparison between NEP-MSC and normal. SEM: standard error of the mean; ANOVA: analysis of variance; A*β*: A*β*_1-42_ injection group; MSC: naïve human umbilical cord-derived mesenchymal stem cell injection group; NEP-MSC: neprilysin-enhanced human umbilical cord-derived mesenchymal stem cell injection group.

To determine the efficacy of naïve and NEP-enhanced hUC-MSCs in AD modeling mice, stem cells were injected twice a week before conducting behavioral experiments (Figure 1). As a result of the Y-maze test, the mice in all groups showed no significant difference in the total number of times they entered the arms of the maze (Figure 3(a)). When the sequences in and out of each arm were analyzed, the mice in the normal group had a high alternation rate in and out of the three arms, but the A*β*_1-42_ injection group showed a low alternation rate (*P*_normal vs.A*β*_ = 0.00002). In contrast, in the naïve and NEP-enhanced hUC-MSC groups, we found that the alternation rate was significantly higher than that in the A*β*_1-42_ injection group, which was not as high as that of the normal group (*P*_A*β* vs.MSC_ = 0.0003 and *P*_A*β* vs.NEP−MSC_ = 0.006). However, there was no difference between the naïve and NEP-enhanced hUC-MSC groups (Figure 3(b)).

The water maze test results showed that from the first day of training, the A*β*_1-42_ injection group differed from the rest of the group in recognizing or remembering the location of the platform, and in subsequent periods, it took longer for the A*β*_1-42_ injection group to find the platform than the rest of the group. In contrast, in the normal group, the time to find the platform decreased during the training period (4 days, *P*_normal vs.A*β*_ = 0.0000002). In the naïve and NEP-enhanced hUC-MSC groups, the time to find the platform was shorter than that in the A*β*_1-42_ injection group, which was less than that in the normal group (4 days, *P*_A*β* vs.MSC_ = 0.0146 and *P*_A*β* vs.NEP−MSC_ = 0.0142) (Figure 3(c)). The results of the probe test on the last day showed that the normal group stayed in the area where the platform was located the longest, and the A*β*_1-42_ injection group tended to wander around the maze regardless of where the platform was located (*P*_normal vs.A*β*_ = 0.000002). The naïve and NEP-enhanced hUC-MSC groups remembered the position of the platform better than the A*β*_1-42_ injection group (*P*_A*β* vs.MSC_ = 0.0060 and *P*_A*β* vs.NEP−MSC_ = 0.0185). However, no memory difference was found between the naïve and NEP-enhanced hUC-MSC groups (Figures 3(d) and 3(e)). This indicated that the naïve and NEP-enhanced hUC-MSC groups in the AD mouse model had improved memory; however, hUC-MSCs with NEP gene modification did not show additional effects in behavioral tests.

### 4.3. Transplantation of Naïve and NEP-Enhanced hUC-MSCs Reduced Amyloid Accumulation in the A*β*_1-42_ Injection Mouse Brain

**Figure 4:**
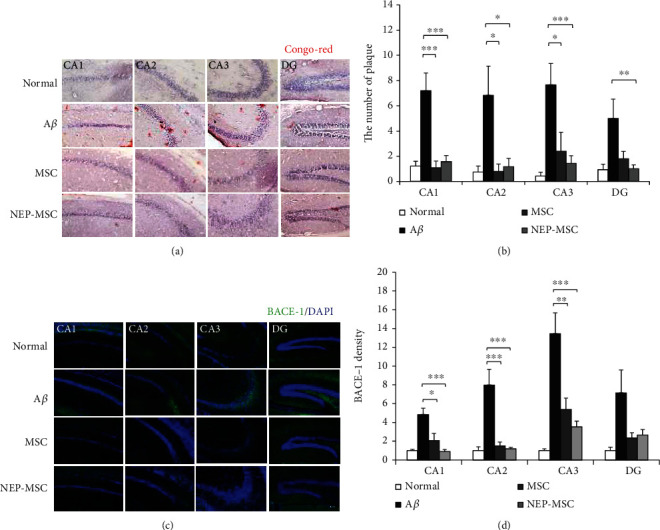
Inhibition of amyloidosis following hUC-MSC transplantation. Representative staining images showing (a) Congo red staining and (c) BACE-1 expression in subregions of hippocampus. Scale bar = 50 *μ*m. The graph shows (b) the number of Congo red stains and (d) density of BACE-1 in each group. The data represent mean ± SEM and were analyzed by one-way ANOVA. ^∗^*P* < 0.05, ^∗∗^*P* < 0.01, and ^∗∗∗^*P* < 0.001 (the marking of *P* values comparing the normal and A*β* groups is omitted from the graph). SEM: standard error of the mean; ANOVA: analysis of variance; hUC-MSC: human umbilical cord-derived mesenchymal stem cell; BACE-1: beta secretase-1; A*β*: A*β*_1-42_ injection group; MSC: naïve hUC-MSC injection group; NEP-MSC: neprilysin-enhanced hUC-MSC injection group; DG: dentate gyrus.

Congo red staining was performed to determine the accumulation status of A*β*_1-42_ in each group. After dyeing with the Congo red reagent, we observed CA1, CA2, CA3, and dentate gyrus (DG) areas of each group of mouse hippocampus and observed that all four sites had more red plaques than normal groups in groups injected with A*β*_1-42_ (normal vs. A*β*: *P*_CA1_ = 0.0001, *P*_CA2_ = 0.0199, *P*_CA3_ = 0.0005, and *P*_DG_ = 0.0059). In contrast, groups injected with hUC-MSCs and NEP-enhanced hUC-MSCs showed a decrease in the amount of these plaques (A*β* vs. MSC: *P*_CA1_ = 0.0001, *P*_CA2_ = 0.0300, and *P*_CA3_ = 0.0227; A*β* vs. NEP-MSC: *P*_CA1_ = 0.0001, *P*_CA2_ = 0.0334, *P*_CA3_ = 0.0024, and *P*_DG_ = 0.0069) (Figures 4(a) and 4(b)). However, the difference in plaques between the naïve and NEP-enhanced hUC-MSC groups was not observed.

### 4.4. Naïve and NEP-Enhanced hUC-MSCs Reduced BACE-1 Expression in the A*β*_1-42_ Injection Mice

To determine the effect of transplantation of naïve and NEP-enhanced hUC-MSCs on BACE-1 protein, the A*β*-producing element, the expression of BACE-1 was assessed by immunohistochemistry in brain samples of each group (Figure 4(c)). In mice injected with A*β*_1-42_, we observed an increase in BACE-1 expression in all areas of CA1, CA2, CA3, and DG of the hippocampus compared to the normal group (normal vs. A*β*: *P*_CA1_ = 0.0018, *P*_CA2_ = 0.0001, *P*_CA3_ = 0.00002, and *P*_DG_ = 0.0267). BACE-1 expression significantly decreased in the CA1, CA2, and CA3 areas of the hippocampus in the naïve and NEP-enhanced hUC-MSC groups compared to the A*β*_1-42_ injection group (A*β* vs. MSC: *P*_CA1_ = 0.0157, *P*_CA2_ = 0.0002, and *P*_CA3_ = 0.0016; A*β* vs. NEP-MSC: *P*_CA1_ = 0.0009, *P*_CA2_ = 0.0001, and *P*_CA3_ = 0.0002) (Figure 4(d)). However, there was no difference in BACE-1 between the naïve and NEP-enhanced hUC-MSC groups.

### 4.5. The Transplantation of Naïve and NEP-Enhanced hUC-MSCs Regenerated BDNF Activity Reduced by A*β*_1-42_

**Figure 5:**
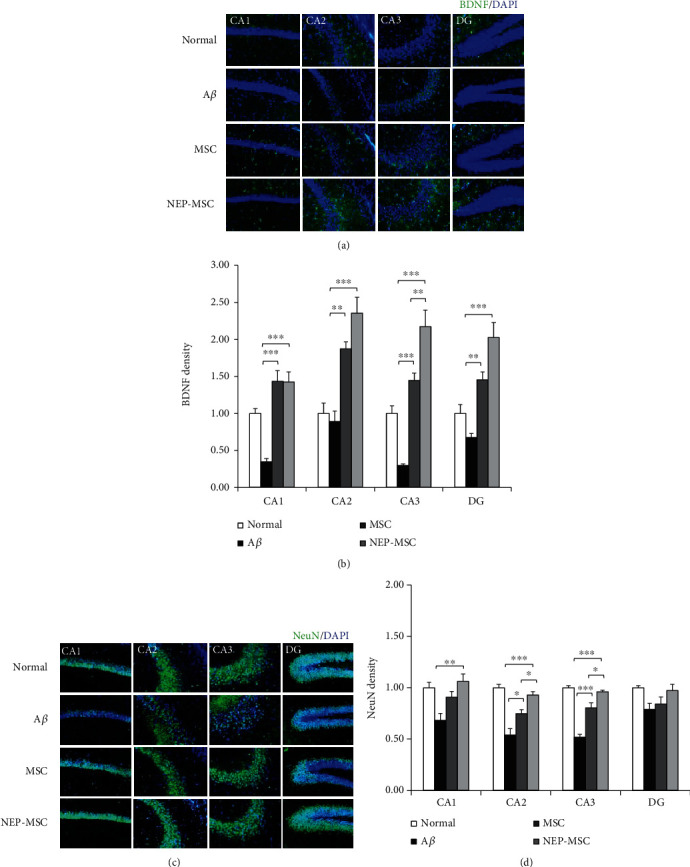
The increase of BDNF expression and neurogenesis following hUC-MSC transplantation. Representative immunostaining images showing (a) BDNF and (c) NeuN expression in subregions of the hippocampus. Scale bar = 50 *μ*m. The graph shows the density of (b) BDNF and (d) NeuN in each group. The data represent mean ± SEM and were analyzed by one-way ANOVA. ^∗^*P* < 0.05, ^∗∗^*P* < 0.01, and ^∗∗∗^*P* < 0.001 (the marking of *P* values comparing the normal and A*β* groups is omitted from the graph). SEM: standard error of the mean; ANOVA: analysis of variance; hUC-MSC: human umbilical cord-derived mesenchymal stem cell; BDNF: brain-derived neurotrophic factor; NeuN: neuronal nuclei; A*β*: A*β*_1-42_ injection group; MSC: naïve hUC-MSC injection group; NEP-MSC: neprilysin-enhanced hUC-MSC injection group; DG: dentate gyrus.

BDNF is an essential element for higher thinking, such as learning and memory, and plays an important role in synaptic plasticity [49]. BDNF protein was dyed and observed in mouse brain samples in order to assess the effect of A*β*_1-42_ injection and transplantation of naïve and NEP-enhanced hUC-MSCs on BDNF expression (Figures 5(a) and 5(b)). As a result, it was observed that the BDNF protein was significantly decreased in the CA1 and CA3 sites of the group injected with A*β*_1-42_ (normal vs. A*β*: *P*_CA1_ = 0.0031 and *P*_CA3_ = 0.0109). In addition, transplantation of naïve and NEP-enhanced hUC-MSCs revealed that the amount of BDNF increased compared to the A*β* group (A*β* vs. MSC: *P*_CA1_ = 0.00001, *P*_CA2_ = 0.0037, *P*_CA3_ = 0.0001, and *P*_DG_ = 0.0087; A*β* vs. NEP-MSC: *P*_CA1_ = 0.00001, *P*_CA2_ = 0.00003, *P*_CA3_ = 0.0000002, and *P*_DG_ = 0.00002). In particular, in the NEP-enhanced hUC-MSC injection group, we observed a significantly higher increase in BDNF levels compared to the naïve group in CA3 (MSC vs. NEP-MSC: *P*_CA3_ = 0.0083).

### 4.6. Naïve and NEP-Enhanced hUC-MSCs Promoted Neurogenesis in the Aß_1-42_ Injection Mice

A*β* accumulation is closely linked to the loss of neuronal cells and synapses in AD [50, 51]. We performed NeuN staining to investigate whether naïve and NEP-enhanced hUC-MSCs contribute to regeneration after the neuronal damage caused by A*β*_1-42_ injection (Figure 5(c)). The expression of NeuN in the A*β*_1-42_ injection group decreased in all areas of CA1, CA2, CA3, and DG in the hippocampus compared to that in the normal group (normal vs. A*β*: *P*_CA1_ = 0.0071, *P*_CA2_ = 0.000003, *P*_CA3_ = 0.000000003, and *P*_DG_ = 0.0451). The expression of NeuN in the naïve and NEP-enhanced hUC-MSC groups significantly increased the expression of NeuN compared to the A*β*_1-42_ injection group (A*β* vs. MSC: *P*_CA2_ = 0.0130 and *P*_CA3_ = 0.00006; A*β* vs. NEP-MSC: *P*_CA1_ = 0.0035, *P*_CA2_ = 0.00004, and *P*_CA3_ = 0.0000003). In addition, the NEP-enhanced hUC-MSC group seemed to have a better nerve cell regeneration effect than the naïve hUC-MSC group (MSC vs. NEP-MSC: *P*_CA2_ = 0.0412 and *P*_CA3_ = 0.0275) (Figure 5(d)). Since the DG is the hub for neurogenesis [24, 52], we focused on the difference in NeuN expression between the hUC-MSC groups. Although NeuN expression in the DG in the NEP-enhanced hUC-MSC group tended to increase compared to that in the naïve group, this difference was not significant. Our results suggest that hUC-MSCs have protective effects against neuronal damage induced by A*β*_1-42_ injection, and that NEP gene modification in hUC-MSCs had additional beneficial effects on neuronal regeneration compared to naïve hUC-MSCs.

### 4.7. Transplantation of Naïve and NEP-Enhanced hUC-MSCs Reduced the Inflammatory Response of the A*β*_1-42_ Injection in the Mouse Brain

**Figure 6:**
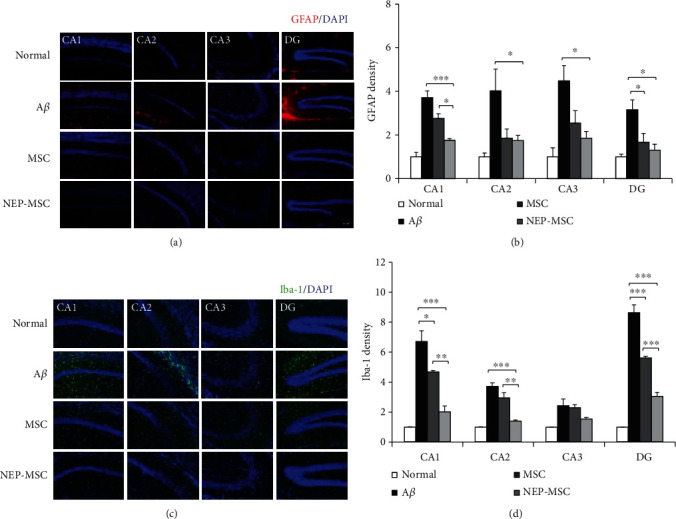
The effects of hUC-MSC transplantation on neuroinflammation. Representative immunostaining images showing (a) GFAP and (c) Iba-1 expression in subregions of the hippocampus. Scale bar = 50 *μ*m. The graph shows the density of (b) GFAP and (d) Iba-1 in each group. The data represent mean ± SEM and were analyzed by one-way ANOVA. ^∗^*P* < 0.05, ^∗∗^*P* < 0.01, and ^∗∗∗^*P* < 0.001 (the marking of *P* values comparing the normal and A*β* groups is omitted from the graph). SEM: standard error of the mean; ANOVA: analysis of variance; hUC-MSC: human umbilical cord-derived mesenchymal stem cell; GFAP: glial fibrillary acidic protein; Iba-1: ionized calcium-binding adapter molecule-1; A*β*: A*β*1-42 injection group; MSC: naïve hUC-MSC injection group; NEP-MSC: neprilysin-enhanced hUC-MSC injection group; DG: dentate gyrus.

In AD, astrocytes and microglia cells are activated and contribute to the development of degenerative dementia [24, 53–57]. In order to investigate whether the transplantation of naïve and NEP-enhanced hUC-MSCs was effective against the inflammation caused by the accumulation of A*β*_1-42_, the brain samples of each group were stained with GFAP as an astrocyte marker and Iba-1 as microglia marker [46] (Figure 6). We observed that the A*β*_1-42_ injection group showed increased GFAP and Iba-1 expression in all areas of CA1, CA2, CA3, and DG in the hippocampus compared to the normal group (GFAP, normal vs. A*β*: *P*_CA1_ = 0.000001, *P*_CA2_ = 0.0045, *P*_CA3_ = 0.0018, and *P*_DG_ = 0.0043; Iba-1, normal vs. A*β*: *P*_CA1_ = 0.000003, *P*_CA2_ = 0.000005, *P*_CA3_ = 0.007, and *P*_DG_ = 0.000000002). Naïve and NEP-enhanced hUC-MSC groups exhibited decreased GFAP and Iba-1 expression compared to the A*β*_1-42_ injection group (GFAP, A*β* vs. MSC: *P*_DG_ = 0.0409; A*β* vs. NEP-MSC: *P*_CA1_ = 0.0001, *P*_CA2_ = 0.0494, *P*_CA3_ = 0.0177, and *P*_DG_ = 0.0118; Iba-1, A*β* vs. MSC: *P*_CA1_ = 0.0276 and *P*_DG_ = 0.00006; A*β* vs. NEP-MSC: *P*_CA1_ = 0.00002, *P*_CA2_ = 0.00003, and *P*_DG_ = 0.00000008). The NEP-enhanced hUC-MSC group showed significantly reduced expression of GFAP and Iba-1 in subregions of the hippocampus compared to the naïve hUC-MSC group (MSC vs. NEP-MSC: GFAP, *P*_CA1_ = 0.0390; Iba-1, *P*_CA1_ = 0.004, *P*_CA2_ = 0.001, and *P*_DG_ = 0.0003) (Figures 6(b) and 6(d)).

### 4.8. Transplantation of NEP-Enhanced hUC-MSCs Increased NEP Expression in A*β*_1-42_-Injected AD Mouse Brains

**Figure 7:**
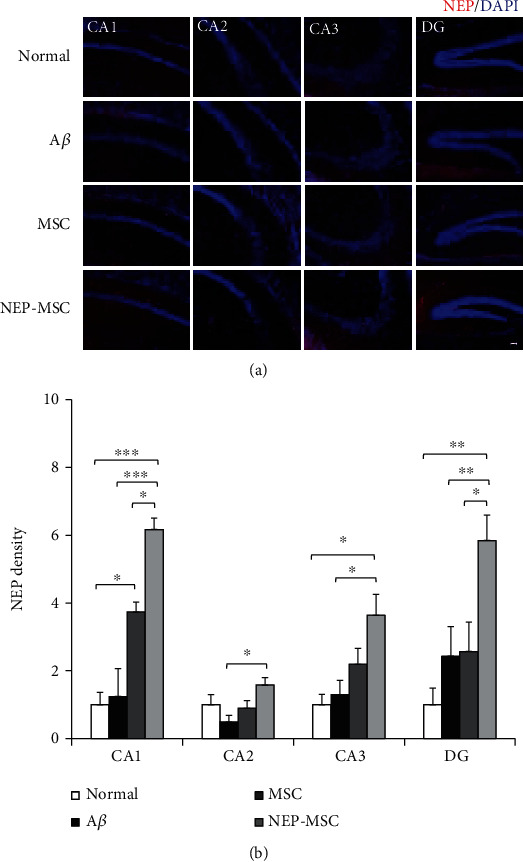
Increased NEP gene expression within brain after transplantation of hUC-MSC with NEP gene transfection. (a) NEP expression in subregions of the hippocampus after injection of naïve and NEP-enhanced hUC-MSCs. Scale bar = 50 *μ*m. (b) The graph shows the density of NEP in each group. The data represent mean ± SEM and were analyzed by one-way ANOVA. ^∗^*P* < 0.05, ^∗∗^*P* < 0.01, and ^∗∗∗^*P* < 0.001 (the marking of *P* values comparing the normal and A*β* groups is omitted from the graph). SEM: standard error of the mean; ANOVA: analysis of variance; NEP: neprilysin; hUC-MSC: human umbilical cord-derived mesenchymal stem cell; A*β*: A*β*_1-42_ injection group; MSC: naïve hUC-MSC injection group; NEP-MSC: neprilysin-enhanced hUC-MSC injection group; DG: dentate gyrus.

To investigate whether transplantation of NEP-enhanced hUC-MSCs actually led to an increase in the expression of NEP in mouse brains, we performed NEP staining. As a result, the A*β*_1-42_ injection group had similar expression levels of NEP to the normal group. The naïve hUC-MSC group tended to have increased levels of NEP protein compared to the normal and A*β*_1-42_ injection groups; however, there was no significant difference between the three groups except in the CA1 area (noraml vs. MSC: *P*_CA1_ = 0.0479) (Figure 7). However, the NEP-enhanced hUC-MSC group had significantly increased expression of NEP compared to other groups, including the naïve hUC-MSC group (NEP-MSC vs. normal: *P*_CA1_ = 0.0001, *P*_CA3_ = 0.0124, and *P*_DG_ = 0.0017; NEP-MSC vs. A*β*: *P*_CA1_ = 0.0002, *P*_CA2_ = 0.0375, *P*_CA3_ = 0.0282, and *P*_DG_ = 0.0026; NEP-MSC vs. MSC: *P*_CA1_ = 0.0479 and *P*_DG_ = 0.0350).

### 4.9. The Effects for Transplantation of Naïve and NEP-Enhanced hUC-MSCs Occurred via Migration of the EVs into the Mouse Brain

**Figure 8:**
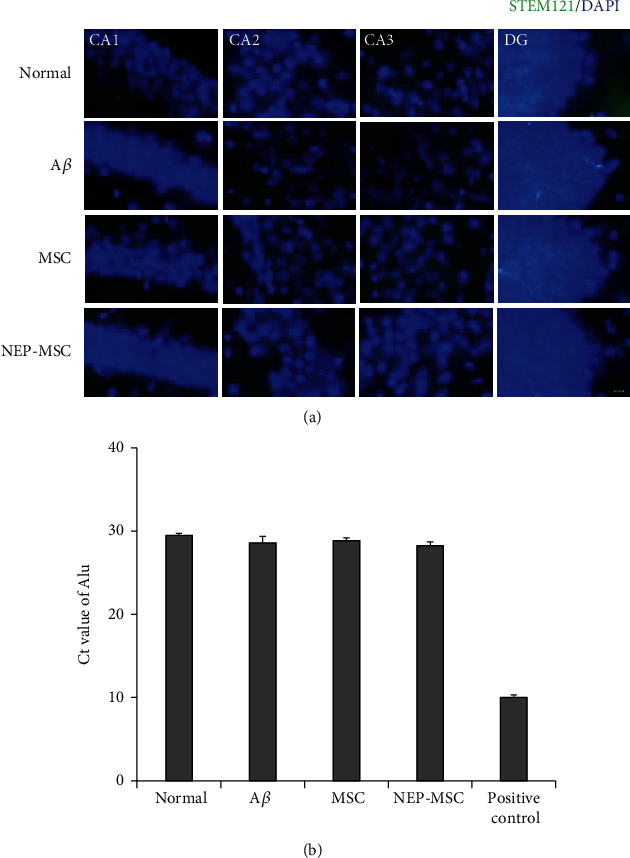
Identification of migration of intravenously transplanted hUC-MSCs. (a) Intravenously transplanted hUC-MSCs (STEM121+ cells) were not detected within brain in all groups. Scale bar = 10 *μ*m. (b) The results of qRT-PCR to investigate the presence of human-specific gene Alu sequences in mouse brain tissues. Positive control used hUC-MSCs. The Alu expression in each group is represented by the Ct value. The data represent mean ± SEM. SEM: standard error of the mean; hUC-MSC: human umbilical cord-derived mesenchymal stem cell; A*β*: A*β*_1-42_ injection group; MSC: naïve hUC-MSC injection group; NEP-MSC: neprilysin-enhanced hUC-MSC injection group; DG: dentate gyrus; qRT-PCR: quantitative real-time polymerase chain reaction.

**Figure 9:**
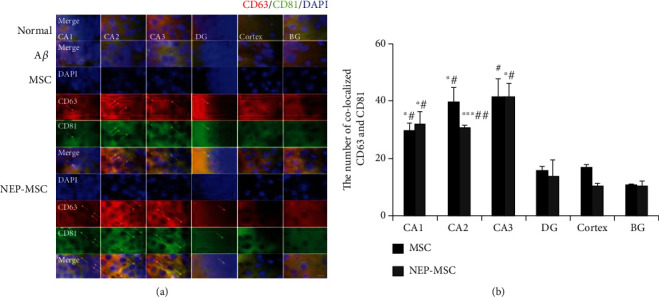
Identification of migration of hUC-MSC-released EVs. (a) Representative image showing colocalization of CD63 and CD81 (human specific)-positive hUC-MSC-released EVs in subregions of the hippocampus. Scale bar = 10 *μ*m. (b) The graph shows the number of colocalized CD63 and CD81-positive hUC-MSC-released EVs in each group. CD63 and CD81-positive EVs were not detected in normal and A*β*_1-42_ injection groups, but only detected in naïve and NEP-enhanced hUC-MSC groups. EVs mainly migrated to the hippocampus than to the cortex and basal ganglia. The data represent mean ± SEM and were analyzed by a *t*-test. ^∗^*P* < 0.05 and ^∗∗^*P* < 0.01 comparison between hippocampus subregion and cortex; ^#^*P* < 0.05 and ^##^*P* < 0.01 comparison between hippocampus subregion and BG. SEM: standard error of the mean; hUC-MSC: human umbilical cord-derived mesenchymal stem cell; A*β*: A*β*_1-42_ injection group; MSC: naïve hUC-MSC injection group; NEP-MSC: neprilysin-enhanced hUC-MSC injection group; DG: dentate gyrus; BG: basal ganglia.

The human cytoplasmic marker (STEM121) was used to determine whether intravenous naïve and NEP-enhanced hUC-MSCs actually reached the mouse brains. No hUC-MSCs were observed in whole mouse brain including hippocampus in any of the groups (Figure 8(a)). Regarding the results of qRT-PCR for the detection of human-specific Alu sequences in each group, the Ct values of naïve and NEP-enhanced hUC-MSC groups were not different from the normal and A*β*_1-42_ injection groups (negative control) (Figure 8(b)). A standard curve in mixed populations was created by converting the dilution percentage to log value. Subsequently, we generated the equation to calculate the number of human cells in mouse brain based on the Ct value. The human cells were detected only in the positive control group, and mouse brain tissue in each group did not contain any human cells (Supplementary Figure 1). However, after dyeing and observing human EV (exosomes) markers CD63 and CD81, we were able to observe EVs within the brain parenchyma in naïve and NEP-enhanced hUC-MSC groups, unlike normal or A*β*_1-42_ injection groups (Figure 9(a)). By counting the number of colocalized CD63 and CD81, we found that similar amounts of these EVs existed in the naïve and NEP-enhanced hUC-MSC groups. In particular, these EVs migrated significantly more to the hippocampus than other cortex and basal ganglia areas (MSC: *P*_CA1 vs.cortex_ = 0.0230, *P*_CA2 vs.cortex_ = 0.0375, *P*_CA1 vs.BG_ = 0.0170, *P*_CA2 vs.BG_ = 0.0278, and *P*_CA3 vs.BG_ = 0.0398; NEP-MSC: *P*_CA1 vs.cortex_ = 0.0338, *P*_CA2 vs.cortex_ = 0.00008, *P*_CA3 vs.cortex_ = 0.0193, *P*_CA1 vs.BG_ = 0.0245, *P*_CA2 vs.BG_ = 0.0027, and *P*_CA3 vs.BG_ = 0.0129) (Figure 9(b)). These results suggest that intravenously administrated hUC-MSCs do not migrate to the brain, while stem cell-released EVs pass the BBB and arrive to the brain parenchyma.

### 4.10. EVs of NEP-Enhanced hUC-MSCs Contain Higher NEP mRNA and Protein Levels than Those of Naïve hUC-MSCs

**Figure 10:**
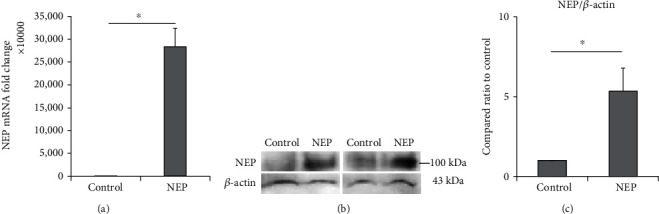
The change of NEP gene expression and protein level in hUC-MSC-derived EVs. (a) The relative amount of NEP mRNA expression in naïve and NEP-enhanced hUC-MSC-derived EVs by the qRT-PCR study. (b) The results of western blot analysis and (c) the amount of NEP protein in naïve and NEP-enhanced hUC-MSC-derived EVs. NEP bands were quantified and normalized against those of *β*-actin. The bar on the graph represents the mean ± SEM, and data were analyzed by a *t*-test. ^∗^*P* < 0.05. SEM: standard error of the mean; hUC-MSC: human umbilical cord-derived mesenchymal stem cell; A*β*: A*β*_1-42_ injection group; MSC: naïve hUC-MSC injection group; NEP-MSC: neprilysin-enhanced hUC-MSC injection group; qRT-PCR: quantitative real-time polymerase chain reaction.

To determine why, although similar numbers of EVs migrated into the brain in both hUC-MSC groups, the NEP-enhanced hUC-MSC group showed a greater tendency for immunohistological effects than naïve hUC-MSCs, we analyzed the amount of NEP within EVs produced by naïve and NEP-enhanced hUC-MSCs by qRT-PCR and western blotting. As a result, EVs derived from NEP-enhanced hUC-MSCs contained significantly higher NEP mRNA and protein levels compared to EVs derived from naïve hUC-MSCs (Figure 10). These results suggest that the increased effects in immunohistological tests after transplantation of NEP-enhanced hUC-MSCs are induced by the increased amount of NEP in stem cell-released EVs.

## 5. Discussion

Our study demonstrated that both naïve and NEP-enhanced hUC-MSCs induced the improvement of cognitive function in A*β*_1-42_-injected mouse AD models, but there was no significant difference between the two groups. In addition, both hUC-MSCs significantly decreased amyloidosis and neuroinflammation and increased BDNF and neurogenesis, which were induced by stem cell-released EVs. The effects on BDNF, NeuN, GFAP, Iba-1, and NEP were significantly more prominent in the NEP-enhanced hUC-MSC group than in the naïve group; this resulted from the increased amount of NEP in the hippocampus from enriched NEP-possessing EVs.

It has been reported that MSCs of various origins, such as the adipose, bone marrow, placenta, and umbilical cord blood, affect functional recovery in AD animal models [58]. Additionally, it has been reported that umbilical cord-derived MSCs improve cognitive function in a transgenic mouse model [12, 24, 59]. hUC-MSCs represent a promising source of MSCs because they can be minimally invasively obtained, easily expanded, not ethically restricted, and have multilineage potential, immunoregulation capacity, and escape from immune surveillance [24, 59]. Therefore, we selected hUC-MSCs as a source of stem cell treatment for AD. As intraparenchymal injection of stem cells is considered high-risk [60] and intra-arterial injection of stem cells causes microembolism [61], we intravenously administered hUC-MSCs [58] to decrease these possible side effects.

In rodent AD models, MSC transplantation has been reported to reduce A*β* deposits/plaque formation [11, 62–64] and tau hyperphosphorylation [46, 65], inhibit A*β*- and tau-related cell death, decrease oxidative stress [12, 59] and neuronal apoptosis [12], stimulate autolysosome formation [46], angiogenesis, neurogenesis, synaptogenesis, and neuronal differentiation [12, 62, 66, 67], and improve spatial learning and memory deficits [63, 65]. Neurogenesis occurs normally in the hippocampus of the adult rat brain [2, 68, 69], and changes in the microenvironment of transplanted MSCs can increase neurogenesis. Our results demonstrated that both naïve and NEP-enhanced hUC-MSC groups exhibited significantly improved cognitive impairment, decreased amyloid plaque and BACE-1, and increased neurogenesis compared to the A*β* injection group.

Although there are few studies supporting neuronal replacement by transplanted stem cells as a therapeutic mechanism, a larger number of studies have reported that this neuroprotection offered by transplanted cells is mediated by neurotrophic factors [3, 70]. A*β* inhibits the activity of the cell signaling pathway of cAMP/PKA/CRE in the hippocampus and downregulates the expression of BDNF [2, 71]. After transplantation of stem cells, the interaction between MSCs and resident brain cells stimulates the production of neurotrophins such as BDNF, vascular endothelial growth factor, nerve growth factor, and anti-inflammatory cytokines, which can potentialize neuritic growth, neuroregeneration, and neurologic recovery [10, 12, 72–75]. Our results revealed that the decrease in BDNF after A*β* injection was significantly increased by transplantation of both hUC-MSCs. Therefore, hUC-MSCs have the potential to reverse A*β*-induced cognitive impairment through a neuroprotective mechanism mediated by BDNF [3, 7].

Regardless of whether AD is initiated by external environmental factors or internal genetic factors, all pathogenic characteristics of AD are accompanied by inflammation [76–78]. It has been reported that accumulated A*β* in AD causes microglial activation and astrocyte recruitment and generates free radicals, nitric oxide, and proinflammatory cytokines [24, 53, 54]. These abnormally activated neuroinflammatory responses could induce BACE-1 expression, facilitate A*β* deposition, exacerbate neurofibrillary tangles, and lead to neuronal damage [24, 55–57]. Inflammation-related proteins, complement factors, and immunoglobulins were found in the senile plaques of patients with AD [76, 79]. MSCs are most notable for their strong immunomodulatory capacity [24, 46, 76]. Some studies have suggested that transplanted MSCs upregulate anti-inflammatory cytokines such as interleukin- (IL-) 10 and reduce the levels of proinflammatory cytokine tumor necrosis factor-*α* and IL-1*β* [63, 65]. In our results, we found that neuroinflammation was significantly decreased in both hUC-MSC groups compared to the A*β* injection group. These results are consistent with those of previous reports [24, 46]. Therefore, it is suggested that this decrease in neuroinflammation could induce decreased amyloidosis and increased memory function in this study.

It has been reported that NEP levels and activity decrease during AD progression [80], suggesting that a reduction in A*β* degradation may contribute to the development of the disease [32, 81–83]. Knockout of NEP in mice results in an increase in the levels of soluble and oligomeric A*β*, leading to impaired synaptic plasticity and cognitive abnormalities in amyloid precursor protein (APP) transgenic and wild-type animals [36, 84–86]. Conversely, overexpression of NEP in AD mouse models using genetic, viral, and pharmacological approaches leads to decreased cerebral A*β* levels [28, 87], inhibition of plaque formation [80], and enhanced cognitive function [88] and life expectancy [30, 36, 40, 89–92]. In line with this, improved behavioral performance and cognitive functions in NEP-overexpressing animals have been reported [36, 92–95]. However, NEP is unable to cross the BBB and requires brain delivery strategies, such as the homing effects of MSCs [96, 97].

To improve the therapeutic effects of MSCs in AD, we manufactured NEP-enhanced hUC-MSCs and transplanted them into A*β*_1-42_-injected mouse AD models. Previous reports have shown that NEP is ubiquitously expressed in brain regions relevant to amyloid accumulation, such as the hippocampus and association cortex, which may participate in regulating neuropeptide signaling [33, 82, 98]. MSCs have been reported to express NEP and increase the clearance of A*β* [46]. In our results, the expression of NEP increased after transplantation of both hUC-MSCs. However, the expression of NEP was significantly increased in the NEP-enhanced hUC-MSC group compared to the naïve group in the hippocampus. In our study, we found that the NEP-enhanced hUC-MSC group had increased levels of BDNF and NeuN and decreased levels of GFAP and Iba-1 compared to naïve hUC-MSCs. Therefore, we suggest that the increased NEP within the hippocampus by NEP-enhanced hUC-MSCs has additional effects on increased neurogenesis and decreased neuroinflammation, compared to naïve hUC-MSCs. Previously, it has been reported that NEP-NSCs reduced A*β* pathology, increased synaptic density, and enhanced the memory and cognitive functions in transgenic mice [9, 14]. In addition, NEP-transfected CD11b+monocytes in AD transgenic mice migrated into the brain, and A*β* deposition was slowed [9, 99]. Also, intranasally administrated EV-loaded NEP in an AD rat model improved the behavioral function through the regulation of inflammation and apoptosis [49, 97]. In our study, we did not find that NEP-enhanced hUC-MSCs had superior effects on cognitive improvement and amyloidosis compared to naïve hUC-MSCs. As we used the gene of membrane-bound NEP in this study, it is possible that we could not detect these additional effects in NEP-enhanced hUC-MSCs compared to naïve hUC-MSCs. Meilandt et al. [100] found a 50% reduction in soluble A*β* levels and prevention of plaque formation [36], but no improvement in cognition in hAPP/NEP double transgenic animals with NEP overexpression [9, 100]. They explained that major differences in delivery approach and the use of secreted versus membrane-bound forms of NEP likely account for these differences [9]. Therefore, further study after changes from the membrane-bound form to the soluble form of NEP may be useful.

Some studies have verified that MSCs can cross the BBB via intravenous transplantation [59, 101, 102]. However, as the size of MSCs is too large, it is difficult for MSCs to pass through the BBB. When MSCs are intravenously administered, they are mostly taken up by peripheral organs such as the liver, lungs, and spleen. Only below 2% of MSCs could migrate into the brain parenchyma, or there was no migration at all. Therefore, it is unlikely that the migration of MSCs into the brain parenchyma contributes to cognitive functional improvement. In our study, we could not find migrating hUC-MSCs over the whole brain. However, we found EVs in the brain parenchyma after transplantation of both hUC-MSCs. Recently, it has been reported that transplanted MSCs influenced AD via their paracrine effects [67, 103–106], and EVs (exosomes) secreted by MSCs contribute to their paracrine effects [103, 107–109]. EVs (exosomes) have been shown to be one of the most important components of the secretory activity of MSCs; they display similar effects to those seen in MSCs [10, 11, 110–113]. Many investigators have demonstrated that MSC-derived EVs (exosomes) improve cognitive deficits in animal models of AD [10, 114, 115]. Also, EVs (exosomes) have been reported to increase neurogenesis, angiogenesis, and synaptogenesis [10, 111, 116–119], possess immunoregulatory and neurotrophic abilities, secrete NEP [8, 103, 120], and decrease A*β* levels and plaques [8, 120, 121]. Exosomes derived from MSCs were found to be transported to the hippocampus, which is the central region associated with AD [121]. Moreover, a previous in vitro study showed that MSCs secrete exosomes, carrying enzymatically active NEP, which were transferred into cocultured N2a cells and decrease both secreted and intracellular A*β* levels in the N2a cells [103]. In this study, we found that EVs mainly migrated into the hippocampus compared to other areas. The amount of migrated EVs did not differ between naïve and NEP-enhanced hUC-MSCs. However, NEP expression was significantly increased in the NEP-enhanced hUC-MSC group compared to the naïve hUC-MSC group. Therefore, it is suggested that the increased NEP within the EVs after gene modification of MSCs induced this difference between NEP expression in the hippocampus of both hUC-MSC groups. In addition, we found an increased amount of NEP in EVs extracted from NEP-enhanced hUC-MSCs compared to naïve hUC-MSCs. Therefore, it is suggested that the increased EV-derived NEP, which is released from NEP-enhanced hUC-MSCs, might induce differences in immunohistochemical assays such as BDNF, neurogenesis, and neuroinflammation between the two hUC-MSC groups. The increase of NEP delivery through the EVs using NEP-enhanced hUC-MSCs may be suggested as a safe therapeutic approach in AD treatment because it does not use viral vectors.

In summary, intravenous administration of NEP-enhanced hUC-MSCs significantly decreased amyloidosis and neuroinflammation, increased BDNF, NEP, and neurogenesis, and improved cognitive function in an AD mouse model. It has been previously reported that NEP-expressing NSCs induced 6-month long-lasting reversing memory and overcoming learning deficits [14]. Although we did not evaluate the long-term efficacy of this therapeutic option for AD, our multiple faceted (multifunctional) activities of NEP-enhanced hUC-MSCs could be expected to promote positive effects on cognitive improvement in the mid- and long-term. Intravenous MSC injection is a safe and universally applied technique in clinical practice for various neurological diseases such as stroke and dementia [21, 122]. Also, NEP-enhanced hUC-MSCs used in this study may be a safe therapeutic approach as no viral vectors are involved. Therefore, our findings suggest that intravenous administration of NEP-enhanced hUC-MSCs might be feasible and could effectively restore cognitive function in AD patients.

## 6. Conclusions

Both naïve and NEP-enhanced hUC-MSCs have an effect on AD animal models; this effect is induced by stem cell-released EVs rather than stem cell penetration. Although there was no significant difference in cognitive improvement between the hUC-MSC groups, we demonstrated that NEP-enhanced hUC-MSCs had superior neurogenesis and neuroinflammation properties compared to naïve hUC-MSCs due to the increased NEP in the hippocampus by enriched NEP-possessing EVs compared to naïve cells. NEP-enhanced hUC-MSCs could be used as an increase system of NEP delivery into the brain through the EVs; this could increase the efficacy of MSCs in AD therapy. Thus, the development of NEP gene-modified MSCs may open a novel horizon for therapeutic strategies in AD. However, to improve the functional efficacy of NEP-enhanced hUC-MSCs, further studies are necessary.

## Data Availability

The datasets generated and/or analyzed during the current study are available from the corresponding author upon reasonable request.

## Abbreviations

A*β*: *β*-Amyloid
A*β* group: A*β*_1-42_ injection group
AD: Alzheimer's disease
ANOVA: Analysis of variance
BBB: Blood-brain barrier
BACE-1: Beta secretase-1
BDNF: Brain-derived neurotrophic factor
Ct: Cycle threshold
DG: Dentate gyrus
EV: Extracellular vesicle
GFAP: Glial fibrillary acidic protein
GFP: Green fluorescent protein
hUC-MSC: Human umbilical cord-derived mesenchymal stem cell
Iba-1: Ionized calcium-binding adapter molecule-1
MSC: Mesenchymal stem cell
MSC group: Naïve hUC-MSC injection group
NEP: Neprilysin
NEP-MSC group: NEP-enhanced hUC-MSC injection group
NeuN: Neuronal nuclei
NSCs: Neural stem cells
qRT-PCR: Quantitative real-time polymerase chain reaction
SEM: Standard error of the mean.

## Supplementary Materials

Supplementary Materials Supplementary Figure 1: quantification of human cells within the mouse brain by the standard curve and equation. (a) The Ct value obtained by qRT-PCR in mixed populations through diluting hUC-MSC to normal mouse cells. (b) The standard curve and equation by the Ct value against log value of hUC-MSC dilution fold. (c) The quantification of human cells within brain tissue of each group based on the Ct value by equation. The data represent mean ± SEM. SEM: standard error of the mean; hUC-MSC: human umbilical cord-derived mesenchymal stem cell; A*β*: A*β*_1-42_ injection group; MSC: naïve hUC-MSC injection group; NEP-MSC: neprilysin-enhanced hUC-MSC injection group; qRT-PCR: quantitative real-time polymerase chain reaction.
