# Supplementary material for: Extracellular Vesicles Released from Neprilysin Gene-Modified Human Umbilical Cord-Derived Mesenchymal Stem Cell Enhance Therapeutic Effects in an Alzheimer's Disease Animal Model
Source: Stem Cells Int. 2021 Dec 3;2021:5548630. doi: 10.1155/2021/5548630 (PMC8664527; doi:10.1155/2021/5548630)
Supplement: Supplementary Materials — Supplementary Figure 1: quantification of human cells within the mouse brain by the standard curve and equation. (a) The Ct value obtained by qRT-PCR in mixed populations through diluting hUC-MSC to normal mouse cells. (b) The standard curve and equation by the Ct value against log value of hUC-MSC dilution fold. (c) The quantification of human cells within brain tissue of each group based on the Ct value by equation. The data represent mean ± SEM. SEM: standard error of the mean; hUC-MSC: human umbilical cord-derived mesenchymal stem cell; Aβ: Aβ1-42 injection group; MSC: naïve hUC-MSC injection group; NEP-MSC: neprilysin-enhanced hUC-MSC injection group; qRT-PCR: quantitative real-time polymerase chain reaction. [file 5548630.f1.pdf]

Supplementary figure 1.

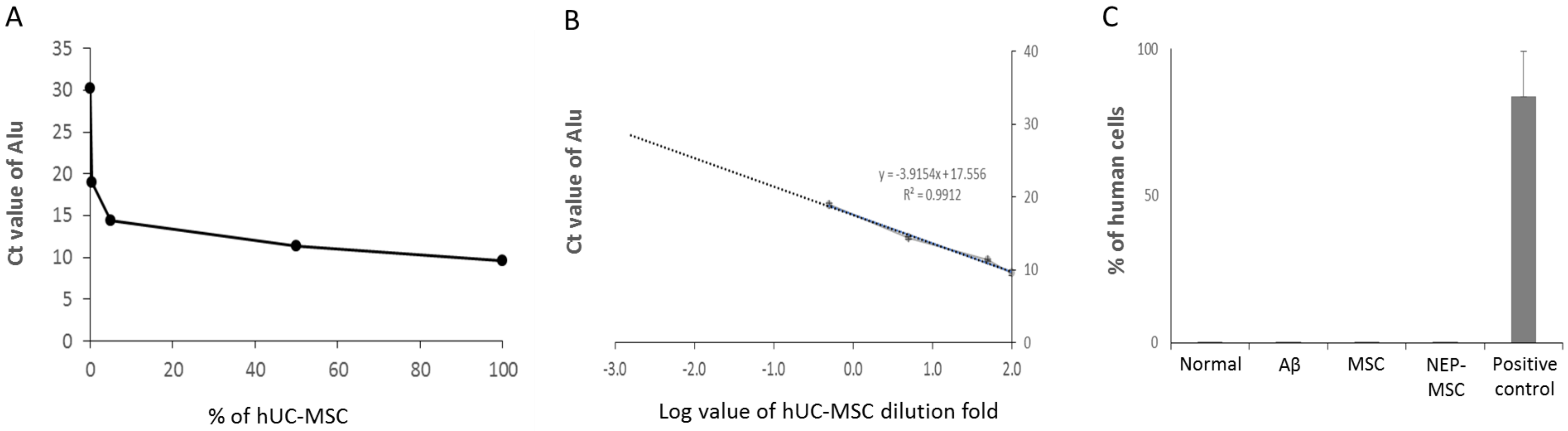

Supplementary figure 1. Quantification of human cells within the mouse brain by the standard curve and equation. (a) The Ct value obtained by qRT-PCR in mixed populations through diluting hUC-MSC to normal mouse cells. (b) The standard curve and equation by the Ct val-ue against log value of hUC-MSC dilution fold. (c) The quantification of human cells within brain tissue of each group based on the Ct value by equation. The data represent mean ± SEM. SEM, standard error of mean; hUC-MSC, human umbilical cord-derived mesenchymal stem cell; Aβ, Aβ1-42 injection group; MSC, naïve hUC-MSCs injection group; NEP-MSC, nepri-lysin enhanced hUC-MSCs injection group; qRT-PCR, quantitative real-time polymerase chain reaction
